# The origin of unwanted editing byproducts in gene editing: Origin of unwanted editing byproducts in gene editing

**DOI:** 10.3724/abbs.2022056

**Published:** 2022-05-25

**Authors:** Jianhang Yin, Jiazhi Hu

**Affiliations:** The MOE Key Laboratory of Cell Proliferation and Differentiation School of Life Sciences Center for Life Sciences Genome Editing Research Center Peking University Beijing 100871 China

**Keywords:** genome editing and CRISPR/Cas, DSB repair, chromosomal translocation, large deletion, vector DNA integration, PEM-seq

## Abstract

The rapid development of CRISPR-Cas genome editing tools has greatly changed the way to conduct research and holds tremendous promise for clinical applications. During genome editing, CRISPR-Cas enzymes induce DNA breaks at the target sites and subsequently the DNA repair pathways are recruited to generate diverse editing outcomes. Besides off-target cleavage, unwanted editing outcomes including chromosomal structural variations and exogenous DNA integrations have recently raised concerns for clinical safety. To eliminate these unwanted editing byproducts, we need to explore the underlying mechanisms for the formation of diverse editing outcomes from the perspective of DNA repair. Here, we describe the involved DNA repair pathways in sealing Cas enzyme-induced DNA double-stranded breaks and discuss the origins and effects of unwanted editing byproducts on genome stability. Furthermore, we propose the potential risk of inhibiting DNA repair pathways to enhance gene editing. The recent combined studies of DNA repair and CRISPR-Cas editing provide a framework for further optimizing genome editing to enhance editing safety.

## Introduction

**Figure 1:**
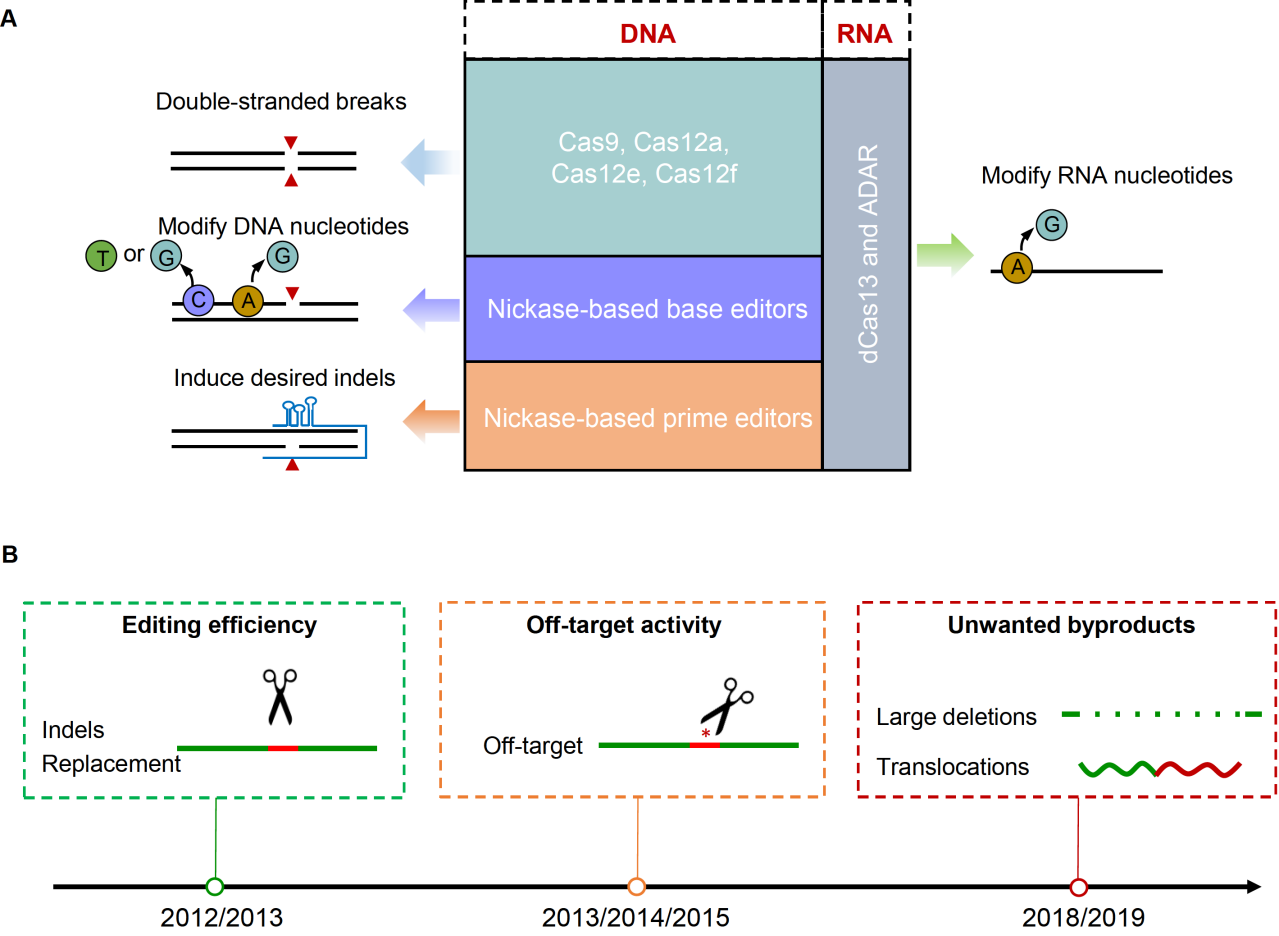
Figure 1 Genome editing tools and the arising concerns (A) Currently used CRISPR-Cas genome editing tools. CRISPR-Cas editing tools can be subtyped into DSB-dependent nucleases, nickase-based base editors, nickase-based prime editors, and dCas13-based RNA editors. Cas9, Cas12a, Cas12e, and Cas12f are widely-used nucleases for genome editing. Base editors can be classified into CBE (C to T), GBE (C to G), and ABE (A to G) based on the conversion or transversion of the nucleotides. Prime editors induce specific insertions and deletions by using an RNA template in the sgRNA scaffold. RNA base editors are designed by fusing dCas13 and ADAR to convert A to G. (B) Concerns in the genome editing field. Editing efficiencies and off-target activities are early concerns in the field. Until recently, unwanted byproducts like large deletions and translocations are appealed by NIH.

The bacterial clustered regularly interspaced short palindromic repeats (CRISPR)-CRISPR-associated protein (Cas) nucleases have been engineered to achieve efficient gene editing in mammalian cells [ 1– 4] . Among the diverse editing toolboxes, Cas nucleases like Cas9, Cas12a, Cas12e, and Cas12f generate DNA double-stranded breaks (DSBs) to initiate gene disruptions ( Figure 1A) [ 5– 10] , while base editors were developed by fusing Cas nickase with a DNA deaminase enzyme to directly modify DNA nucleotides instead of inducing DSBs ( Figure 1A) [ 11– 13] . Of note, the Cas nickase embedded in base editors can generate single-stranded breaks (SSBs) during the base editing process [ 11– 13] . The more recent prime editors consist of a Cas9 nickase and a reverse transcriptase to induce template-dependent insertions or deletions ( Figure 1A) [14]. Regarding RNA editing, ADAR or nuclease-dead Cas13-fused ADAR can modify RNA nucleotides for gene interference ( Figure 1A) [ 15, 16] . The versatile CRISPR-Cas editing system has been widely used in both scientific research and clinical therapeutics. Many clinical trials employing CRISPR-Cas nucleases are underway and the preliminary results are very promising ( Table 1). These CRISPR-based therapeutic schemes target some very intractable diseases, including T cell, chimeric antigen receptor T cell (CAR T), or T cell receptor-engineered T cell (TCR T) therapy for acquired immunodeficiency syndrome (AIDS) and malignancy [ 17– 19] , modified hematopoietic stem and progenitor cells (HSPCs) for transfusion-dependent β-thalassemia and sickle cell disease [ 20, 21] , correcting *CEP290* for Leber Congenital Amaurosis type 10 [22], and Duchenne muscular dystrophy [ 23– 25] .

**Table:** **Table 1** Application of Cas9 nuclease in clinical therapy*

Target gene	Clinical ID	Therapy	Disease	Stage
*TRAC*, *B2M*	NCT03166878, NCT03229876, NCT03166878	CAR-T	B cell leukemia	Phase 1/2
*TRAC*, *TRBC*, *B2M*	NCT04244656, NCT03166878	T cell therapy, CAR-T	Myeloma, B cell lymphoma	–
*TRAC*, *TRBC*, *PDCD1*	NCT03399448, NCT03545815	TCR-T	B cell leukemia and solid tumor	Phase 1
*TRAC*	NCT03398967	CD19, CD20 or CD22 CAR-T therapy	Relapsed or refractory hematological malignancies	Phase 1/2
*PDCD1*	NCT02793856	T cell therapy	Advanced Non-small Cell Lung Cancer	Phase 1
NCT02863913	T cell therapy	Stage IV bladder cancer	Phase 1
NCT02867332	T cell therapy	Metastatic renal cell carcinoma	Phase 1
NCT03081715	T cell therapy	Esophageal cancer	Phase 1
NCT02867345	T cell therapy	Hormone refractory prostate cancer	Phase 1
*CD7*	NCT03690011	CAR-T	T cell leukemia	Phase 1
*CD70*	NCT04438083	T cell therapy	Hematologic malignancies and renal cell carcinoma	Phase 1
*CCR5*	NCT03164135	HSPC therapy	HIV and leukemia	–
*BCL11A*	NCT03432364, NCT03655678, NCT03745287, CRISPR_SCD001	HSC therapy	Transfusion-dependent β-thalassemia (TDT) and sickle cell disease (SCD)	Phase 1/2
*HBB*	NCT03728322	HSC therapy	TDT	Phase 1
*CEP290*	NCT03872479	AAV therapy	Leber congenital amaurosis type 10 (LCA10)	Phase 2
*CISH*	NCT03538613, NCT04089891	T cell therapy	Metastatic gastrointestinal epithelial cancer	Phase 1/2

*Because of space limitation, only typical clinical trials are cited here.

Besides the great potential of CRISPR-Cas editing tools, unwanted editing byproducts accompanied with intended editing products have also attracted great attention recently, since they lend additional uncertainty to genome editing [26]. These unwanted editing byproducts include but are not limited to off-target damages, chromosomal structural variations, and exogenous DNA integrations ( Figure 1B). Many efforts have been made to further improve the performance of CRISPR-Cas gene editing tools [ 27– 29] and various methods based on experiments or in silico prediction have been developed to identify or evaluate off-target activity for Cas nucleases ( Table 2, see below for more details) [ 30– 40] . Chromosomal translocations and large deletions have also been routinely observed in different editing scenarios recently [ 31, 37– 39, 41– 45] . For example, chromosomal abnormalities have been discovered to expand in a patient treated by CAR T cells manufactured by Allogene, which leads to the hold on Allogene CAR T therapeutics. An effective method to reduce chromosomal abnormalities during gene editing is still lacking.

**Table:** **Table 2** Methods for the detection of byproducts generated by Cas9*

Method	*In vivo*/ *In vitro*/ *In silico*		Assay type	Comment
Cas-OFFinder	*In silico*	Off-target	Sequence alignment	High false positive
CAST-seq	*In vivo*	Chromosomal structural variations, indels	Map translocations with induced DSBs	High-sensitivity; not applicable to limited material
CIRCLE-seq	*In vitro*	Off-target	Sequence cleaved linear DNA from circularized genomic DNA	High-sensitivity; Requires *in vivo* cleavage confirmation
Dig-seq	*In vitro*	Off-target	Whole-genome sequencing for cleaved chromatin	High-sensitivity;
Digenome-seq	*In vitro*	Off-target	Whole-genome sequencing for cleaved naked genomic DNA	High-sensitivity; Requires *in vivo* cleavage confirmation
DISCOVER-seq	*In vivo*	Off-target	Pull down Mre11 binding to broken ends	Narrow time-window (only maps unjoined ends); low resolution
GUIDE-seq	*In vivo*	Off-target	Integrate dsODNs into DSB sites	Unbiased; limited use for blunt-ended DSBs
LAM-HTGTS	*In vivo*	Off-target, chromosomal structural variations	Map translocations with induced DSBs or recurrent DSBs	High-sensitivity; not applicable to limited material
PEM-seq	*In vivo*	Off-target, chromosomal structural variations, indels	Map translocations with induced DSBs or recurrent DSBs	High-sensitivity; not applicable to limited material
SITE-seq	*In vitro*	Off-target	Map broken ends with biotinylatedadapters	High-sensitivity; Requires *in vivo* cleavage confirmation
UDiTaS	*In vivo*	Chromosomal structural variations, indels	Map translocations with induced DSBs	Low sensitivity due to no nested PCR

*Because of space limitation, only typical methods are cited here.

The generation of both intended products and unwanted editing byproducts during genome editing are stimulated by endogenous DSB repair pathways, and understanding how these repair pathways work in depth could help to reduce the side effects of unwanted byproducts during gene editing.

In this review, we begin with the editing mechanism for CRISPR/Cas editing system and then describe the involved DSB repair pathways in the editing process. We next discuss the generation of unwanted genome editing products and propose possible solutions to improve the safety of gene editing.

## CRISPR-Cas Induces DNA Breaks to Initiate Gene Editing

**Figure 2:**
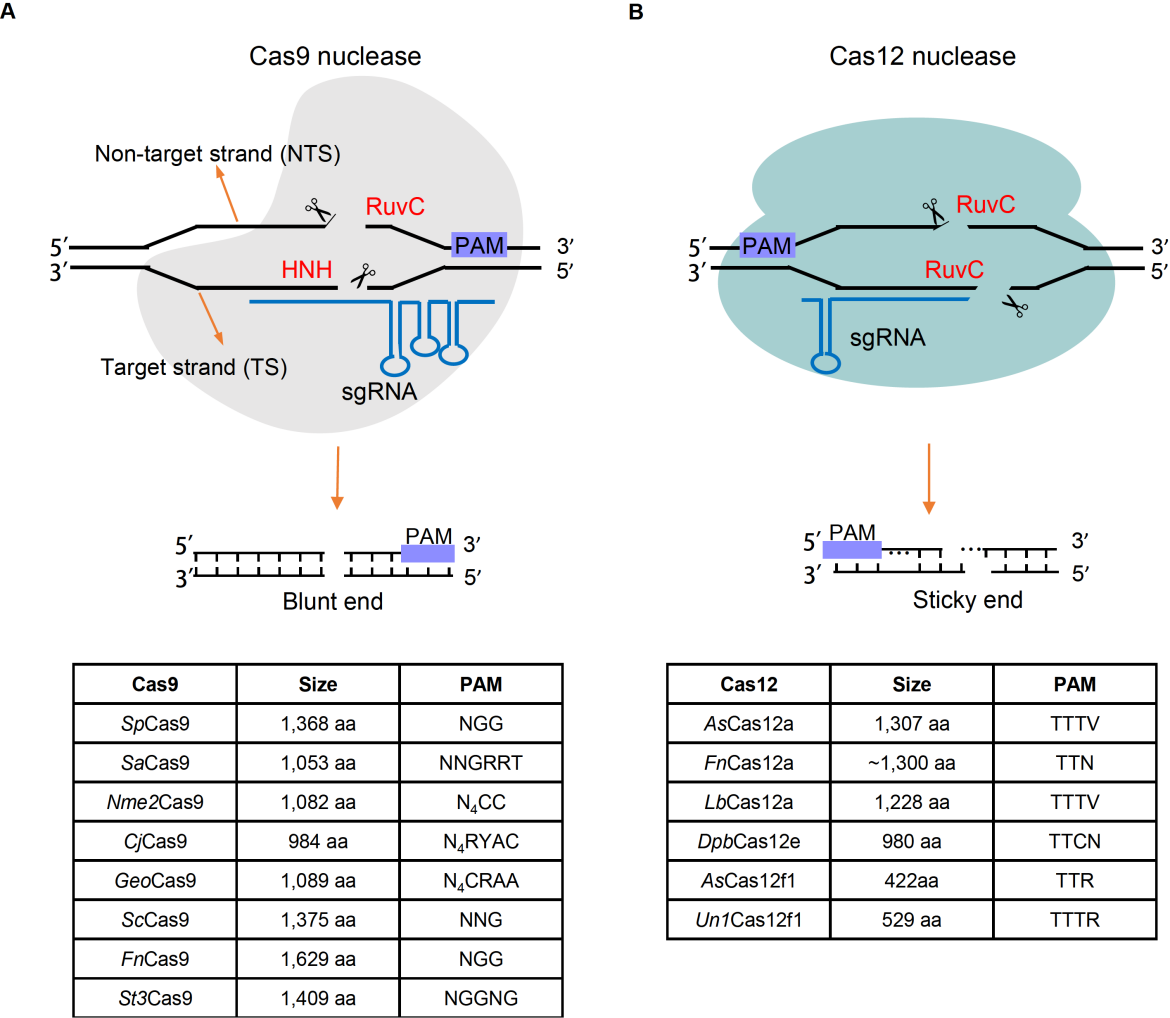
Figure 2 Two main Cas nucleases for genome editing Cas nucleases are guided by sgRNA to the target site by forming an “R loop”. (A) Cas9 nucleases use the HNH domain to cleave the target strand and RuvC domain to cleave non-target strand both upstream PAM. Cas9 nucleases tend to generate blunt ends. (B) Unlike Cas9, Cas12 nucleases use only the RuvC domain to cleave both target and on-target strand downstream PAM, and Cas12 nucleases generate sticky end. Currently-developed Cas9 and Cas12 nucleases with their size and PAM are displayed on the bottom.

The CRISPR-Cas enzyme is an RNA-guided endonuclease that induces DSB at the phage genome. The CRISPR-Cas enzymes have two distinct groups: class I, which applies multi-Cas proteins to achieve DNA cleavage; and class II, which applies a single endonuclease for DNA cleavage [ 46, 47] . Class II is further subtyped into three types: II, V, and VI. The type-II Cas9 recognizing 3′ G rich protospacer adjacent motif (PAM) and type-V Cas12 recognizing 5′ T rich PAM have been engineered for efficient genome editing [48]. Among the engineered Cas9 enzymes, *Streptococcus pyogenes* Cas9 ( *Sp*Cas9) with an NGG (N= “A”, “T”, “C”, or “G”) PAM is the first and most widely used Cas9 for genome editing [ 1– 4, 49] . A smaller size *Staphylococcus aureus* Cas9 ( *Sa*Cas9) was also developed for target sites with NNGRRT (R=“A” or “G”) PAM [50]. Several other Cas9 nucleases including *Streptococcus thermophiles* Cas9 ( *St*Cas9), Campylobacter jejuni Cas9 ( *Cj*Cas9), *Francisella novicida* Cas9 ( *Fn*Cas9), *Geobacillus stearothermophilus* Cas9 ( *Geo*Cas9), *Neisseria Meningitides* Cas9 ( *Nme*Cas9), and *Streptococcus canis* ( *Sc*Cas9) were subsequently engineered for genome editing ( Figure 2A) [ 51– 57] . Regarding the Cas12 family, *Acidaminococcus* sp. Cas12a ( *As*Cas12a) and *Lachnospiraceae* bacterium ND2006 Cas12a ( *Lb*Cas12a) show great potential in gene editing [6]. Recently, orthologs of small-size Cas12e and Cas12f nucleases have been successfully used for gene editing and show advantages for adeno-associated virus (AAV) package for gene therapy ( Figure 2B) [ 7– 10] .

CRISPR-Cas9 system contains a CRISPR RNA (crRNA) for targeting DNA and a *trans*-activating crRNA (tracrRNA) pairing with crRNA for Cas9 ribonucleoprotein (RNP) package [58]. The crRNA and tracrRNA are further combined into a chimeric single guide RNA (sgRNA), which reserves the high cleavage capacity [5]. Cas9 cleavage begins with the recognition for the PAM sequence located at 3′ of the target DNA, followed by the formation of RNA-DNA hybrid (R loop), Cas9 conformation change, and DNA strand cleavage [ 5, 59, 60] . The target strand (pair with sgRNA) is cleaved by the HNH domain and the non-target strand is cleaved by the RuvC domain and both cleavages occur between the third and fourth nucleotides upstream of PAM, which eventually leads to a blunt-ended DSB ( Figure 2A) [5]. Cas9 can also generate 1-bp staggered ends at some target sequences due to the flexible cleavage position of the RuvC domain, resulting in predictable 1-bp insertions [ 43, 61– 64] . Mutation in either of the two cleavage domains generates Cas9 nickase and mutations in both the cleavage domains generate nuclease-dead Cas9 (dCas9) but reserve DNA-binding activity [ 65, 66] . After cleavage, Cas9 nuclease may stay at the PAM-distal ends until the DNA repair proteins are recruited to seal the broken ends [ 59, 67] . In contrast to Cas9 nucleases, most Cas12 nucleases are guided by a single crRNA and equipped with only a RuvC domain to cleave the DNA strands [ 6, 68] . The RuvC domain of Cas12 nuclease cuts the two DNA strands at varied nucleotides and thus results in sticky-ended DSBs ( Figure 2B).

In addition to CRISPR-Cas nucleases, CRISPR-based base editors and prime editors were mainly developed for mutation corrections. The base editor consists of a Cas9 nickase, a DNA deaminase enzyme, and a uracil-DNA glycosylase inhibitor or uracil-DNA glycosylase, which converts C to T, C to G, or A to G without causing DSBs [ 11– 13] . In this context, AID, APOBEC1, APOBEC3A, and APOBEC3B were used as cytosine base editors (CBEs) for C to T conversion or used as glycosylase base editors (GBEs) for C to G transversion [ 11, 13, 69– 71] . In addition, TadA was engineered as an adenine base editor (ABE) for A to G conversion [12]. ABE was further combined with CBE to generate dual base editors for simultaneous C to T and A to G conversions by several research groups [ 72– 74] . Instead of DNA deaminase enzymes, the prime editors fuse a reverse transcriptase with Cas9 nickase to introduce point mutations not covered by CBEs or ABEs, short insertions, and deletions of several nucleotides by using a template RNA within the sgRNA scaffold [14]. The two base editing systems are complementary to Cas9 nucleases for different editing scenarios but also face several problems. First, the Cas9 nickase-induced SSBs can be converted to DSBs at a frequency of 1 out of 100 [ 75, 76] , which may also lead to chromosomal structural variations as Cas9 nucleases though at a low level. Second, base editors have been reported to have collateral DNA and RNA activities besides off-target activities [ 77– 81] .

## DSB Repair Pathways Are Involved in Gene Editing

**Figure 3:**
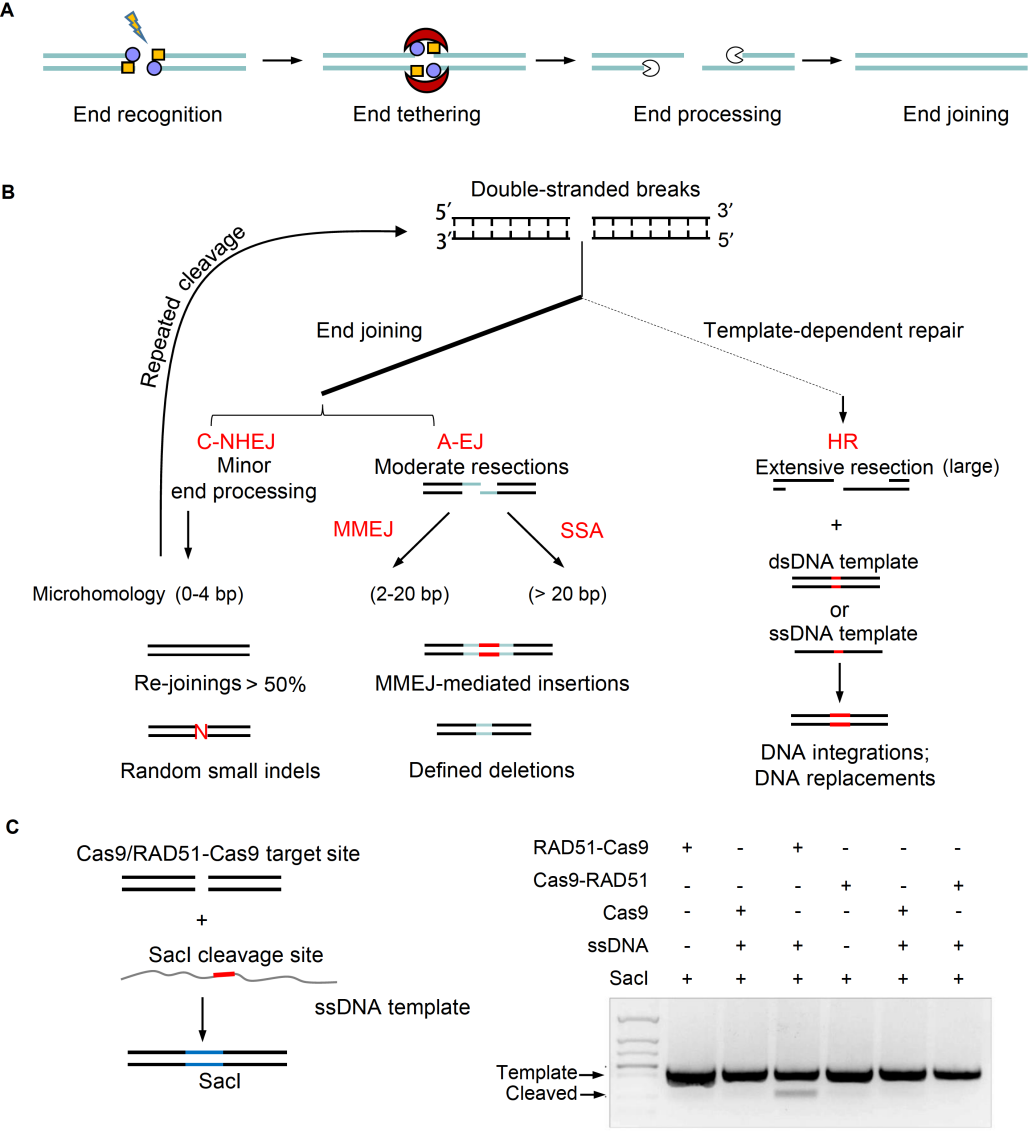
Figure 3 DSB repair pathways involved in genome editing (A) General steps for DSB repair. End recognition, end tethering, end processing, and end joining. (B) DSB repair pathways for genome editing. DSB repairs are mainly subtyped into end-joining and template-dependent repair. C-NHEJ directly joins two broken ends with small indels in the final products due to limited end processing. Note that more than 50% of products generated by C-NHEJ are re-joinings and will undergo several cycles of repeated cleavage until the formation of indels. The process of end-joining may use homology on the broken ends to generate defined deletion or insertions. Due to the length of the homology, homology length from 2 to 20 bp is recognized as MMEJ and more than 20 bp is recognized as SSA. Template-dependent repair needs extensive resection and uses dsDNA template or ssDNA template. (C) RAD51-Cas9 enhances ssDNA integration. ssDNA template with a SacI cleavage site and homologous arm is co-transfected with Cas9 or RAD51-Cas9. If integration occurs, the DNA bands can be cleaved by the restriction enzyme SacI.

DSBs are the most deleterious type of DNA lesions, leading to genetic mutations or complex chromosomal rearrangements associated with oncogenesis [ 75, 82– 87] . Each human cell is subjected to 25 endogenous DSBs per day in estimation [75], and thereby robust DSB repair pathways evolve in mammalian cells to recognize and repair emerging DSBs. Typically, the entire process of DSB repair consists of three or four steps: end recognition, end tethering, end processing if necessary, and end joining ( Figure 3A) [82]. The initial end-recognizing and end-binding proteins determine the choice of the DSB pathways, and then other repair proteins are recruited into the DSBs step-by-step until end joining [ 82, 88– 91] . The mammalian cells mainly evolve two types of DSB repair pathways: template-independent end joining repair and template-dependent homology-directed repair ( Figure 3B). These repair pathways compete with each other and are influenced by cell type, cell state, and the nature of the DSBs [92]. The repair of Cas-induced DSBs shares main features with endogenous DSBs except that Cas9 residence at broken ends may have a weak impact on DSB repair [67]. Here we provided a brief overview of these DSB repair pathways involved in gene editing in mammalian cells.

### Non-homologous end joining

Classical non-homologous end joining (C-NHEJ) directly re-joins two broken ends and is considered to be the default choice for DSB repair in mammalian cells through cell cycles [93]. In estimation, more than 50% of Cas9-induced DSBs are repaired by NHEJ in human pluripotent stem cells or human cell lines within the first 10 h of DSBs [ 67, 94, 95] . C-NHEJ is an error-prone repair process and usually introduces small nucleotide insertions and deletions (indels). Therefore, the CRISPR-Cas targeting at open reading frames can readily induce gene disruption by C-NHEJ-mediated frameshift. However, it is notable that more than 50% of Cas9-induced breaks are perfectly re-joined without end processing in mouse embryonic stem cells (mESCs) and HEK293T cells [ 96, 97] . In this context, the perfectly re-joined products can be targeted repeatedly by CRISPR-Cas enzymes to accumulate desired editing outcomes.

During C-NHEJ, KU70-KU80 heterodimer immediately binds to the broken ends and recruits the DNA-dependent protein kinase catalytic subunit (DNA-PKcs) and/or Artemis endonuclease to mildly process broken ends if needed [ 98– 104] . Next, XRCC4, LIG4, XLF, and recently-identified PAXX proteins are recruited to tether and seal the broken ends [ 105– 112] . Besides Artemis, nucleases such as PALF, MRN complex, and polymerases including the terminal deoxynucleotidyltransferase (TdT), Pol μ, and Pol λ also contribute to the end processing to introduce indels within final products [ 113– 119] . In this context, fusing Cas nucleases with end processing enzymes including T5 and TREX2 facilitates indel formation [ 120, 121] .

### Alternative end joining

Alternative end joining (A-EJ) dominates end joining repair when core factors of C-NHEJ are deficient [ 122, 123] . According to the length of microhomology used, A-EJ can be further divided into two subtypes: microhomology-mediated end joining (MMEJ) with homology at approximately 2–20 bp and single-strand annealing (SSA) pathway which requires large homology (>20 bp) ( Figure 3B) [ 43, 124] . In comparison, C-NHEJ only uses microhomology less than 4 bp ( Figure 3B) [ 43, 124, 125] . A-EJ also functions in the presence of C-NHEJ and competes with C-NHEJ to repair Cas9-induced DSBs [ 126, 127] . By examining over 1000 loci cleaved by Cas9, van Steensel and colleagues recently reported that the choice for MMEJ and C-NHEJ may be influenced by chromatin accessibility and MMEJ tends to occur in heterochromatin regions associated with H3K37me3 modification [127]. Moreover, it has been reported that MMEJ displays delayed activity in comparison with C-NHEJ detected by a quantitative time-course study [67].

MMEJ prevalently contributes to the formation of indels during genome editing by generating short deletions between two microhomologous sequences ( Figure 3B). MMEJ-mediated deletions are relatively predictable in the context of embedded microhomology in local sequence [ 61, 62, 64, 127, 128] . MMEJ enhancement by placing two designed microhomologous sequences spanning the CRISPR-Cas9 target site can efficiently induce programmed fragment insertions and deletions during genome editing [ 129– 132] . SSA is useful for large DNA fragment deletion in genome editing and is mainly active in the S/G2 phase for the need of long exposed homology. Zhang and Matlashewski found that up to 90% of editing products in *Leishmania* were repaired by SSA, and thereby SSA was enhanced to achieve large fragment deletion up to 29 kb [133]. Pol θ, MRN complex and poly (ADP-ribose) polymerase 1 (PARP1) are required in MMEJ [ 134– 137] . SSA shares end resection steps with homologous recombination (HR) to repair DSBs in mammalian cells. For example, CtIP, EXO1, and DNA2 function in both SSA and HR [ 138– 140] .

### Homologous recombination

HR requires a homologous template to finish DSB repair and therefore is a relatively precise DNA repair pathway. HR is mainly active in the S and G2 phases in dividing cells, exhibiting a lower utilization rate in comparison with NHEJ in most cells. The deactivation of C-NHEJ makes Cas-induced DSBs prone to be repaired by A-EJ or HR [ 43, 141– 146] . HR is characterized by extended DNA resection and thereby EXO1 and DNA2 responsible for long-distance DNA resection are critical for HR [ 138, 147– 149] . The highly-processed broken ends are then protected by RPA, followed by RAD51-mediated strand invasion and polymerase-mediated fill-in [ 150– 152] . Recently, RNA polymerase III was also reported to function in HR and protect the processed DNA ends [153].

To introduce intended mutations at target sites during gene editing, a double-stranded (ds) or single-stranded (ss) donor DNA is transfected with CRISPR-Cas to activate the HR pathway to induce homology-directed repair (HDR) ( Figure 3B) [ 154, 155] . The homologous sequence for dsDNA donors is usually hundreds in length while the length of the homologous sequence for ssDNA can be as short as dozens of nucleotides [156]. HDR with ssDNA donor is more frequently used for gene editing, due to moderate adverse cellular responses such as avoiding cGAS activation [157]. Given that HDR is at such a low usage rate, inhibitors for C-NHEJ core factors have been used to enhance HDR during gene editing. For example, the small molecule inhibitors 5102 and 5135 were applied to enhance HDR at a 6-fold increase by suppressing the DNA-binding activity of the KU70/KU80 complex [158]. And the inhibitors of DNA-PKcs, NU7026, and KU-0060648 were used to enhance the HDR by 3 folds [143]. Moreover, applying SCR7 to inhibit the LIG4 showed an increase of 5- to 19-fold for HDR usage in mammalian cell lines [ 141, 142] . In addition to C-NHEJ inhibitors, a dominant-negative form of 53BP1 was expressed with CRISPR-Cas9 to enhance HDR frequency up to 86% in various human cell types [159]. Besides suppression of C-NHEJ, stimulating HR can also enhance HDR. In this context, small molecule RS-1, by activating RAD51, could improve HDR usage up to 5 folds in rabbit embryos [160]. Alternatively, fused or co-expressed RAD51 with Cas9 could also improve HDR ( Figure 3C) [ 161– 164] . In addition, a fusion of truncated CtIP and Cas9 also showed at least 2-fold enhancement for HDR in human cell lines, pluripotent stem cells, and rat zygotes [165]. Furthermore, Chin and colleagues fused human GEMININ to the N terminal of Cas9 to specifically express Cas9 in the S/G2/M phase and increased the rate of HDR by up to 87% [166]. Moreover, arresting cells in S or G2/M phase or inhibiting mismatch repair (MMR) has also been reported to enhance single-stranded DNA oligonucleotide-mediated integration for gene editing [ 167– 172] .

## Unwanted Editing Byproducts of CRISPR-Cas Increase Genome Instability

**Figure 4:**
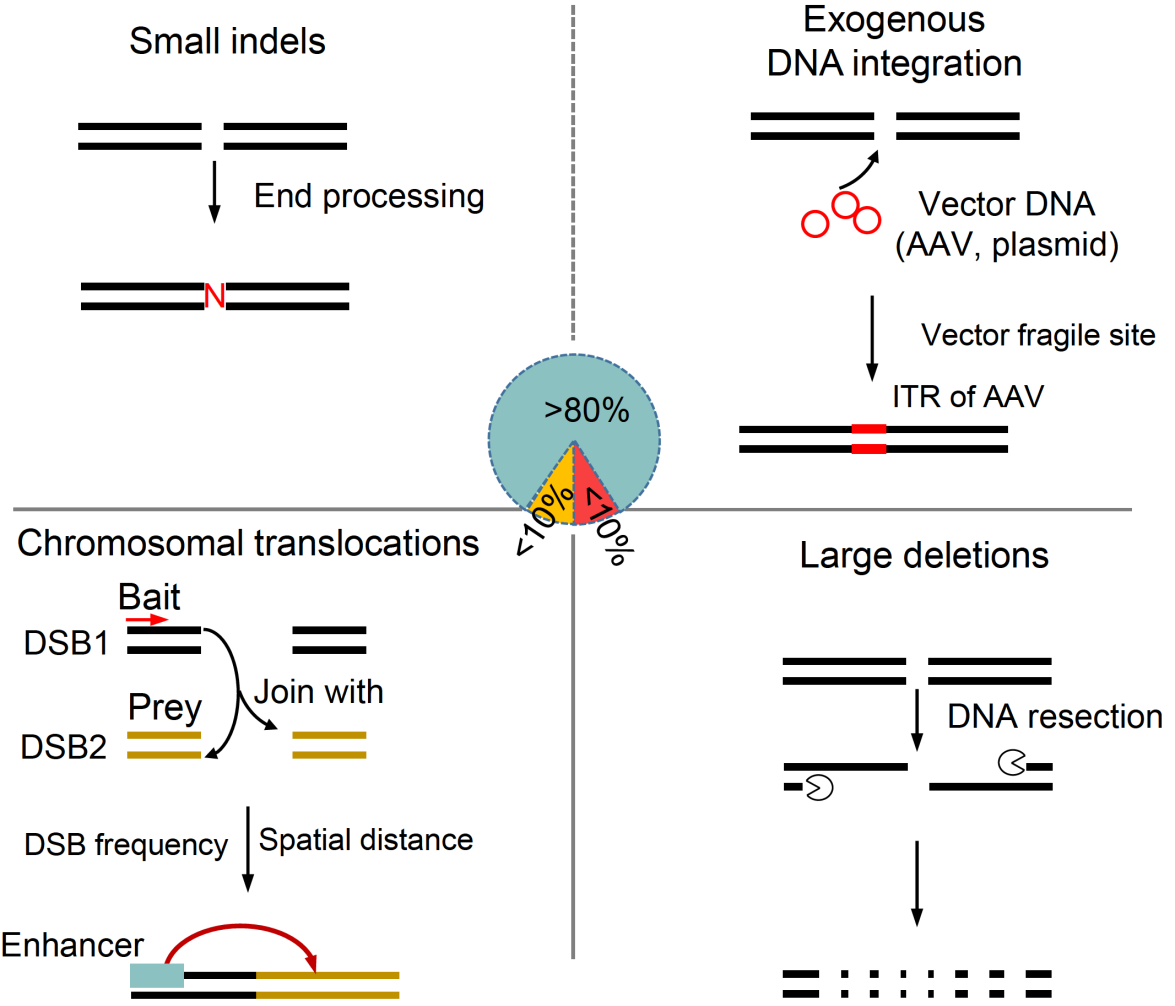
Figure 4 Products and byproducts generated during genome editing In addition to small indels (top left), vector DNA from plasmid or virus can be integrated into the target site. ITR elements from AAV have been reported to function as enhancers (top right). The juxtaposition of two DSBs forms chromosomal translocations. Both DSBs frequency and spatial distance contribute to the translocation formation (bottom left). Large deletions are generated by end-joining after extensive DNA resection at the target site (bottom right).

Given that the repair of Cas9-induced DSBs is consistent with the repair of endogenous general DSBs, it is inevitable that the sealing of Cas9-induced DSBs results in many diverse outcomes. Besides the intended mutations at the target site, other unwanted byproducts are routinely identified. CRISPR-Cas activities at off-target sites are well explored by developed methods and a dozen of high-fidelity Cas9 variants have been engineered to reduce the off-target activities of CRISPR-Cas9 [ 32, 34– 38, 173– 179] . Chromosomal structural variations such as chromosomal translocations and large deletions have also attracted great attention recently, which may cause genome instability and have pathogenic consequences [ 37, 38, 41– 45] . Furthermore, vector integrations are also frequently detected when AAV or other DNA-based delivery methods are used ( Figure 4) [ 44, 180, 181] . In this section, we will discuss the mechanism underlying the unwanted editing byproducts and summarize the currently used methods for the detection of unwanted editing byproducts.

### Off-target activities

Off-target sites of CRISPR-Cas9 are highly homologous to the target sites with higher mutation tolerance at the PAM-distal region [182]. The seed sequence in the target DNA (10–12 nucleotides located in the 3 prime ends of the 20-nt sgRNA) is vital for Cas9 cleavage, and mutations in the seed region nearly block Cas9 cleavage, yet mutations in other regions cause off-target cleavage [ 183– 185] . To improve the specificity of Cas proteins, a dozen of high-fidelity variants have been developed to obtain lower off-target activities. e *Sp*Cas9(1.1), *Sp*Cas9-HF1, and HypaCas9 were developed based on Cas9-DNA structures and Cas9 conformation change before cleavage [ 177– 179] . Sniper-Cas9, evo-Cas9, and xCas9 were developed by high-throughput screening methods [ 174, 176, 186] . These high-fidelity variants perform well at some target loci, however, the sacrifice of the editing efficiency was also detected at certain loci for some variants [ 44, 173] . Moreover, because chromosomal structural variations are byproducts during the process of DSB repair and mainly occur at the target sites, high-fidelity variants could not reduce chromosomal translocations and large deletions caused by Cas nuclease [44].

### Chromosomal translocations

The juxtaposition of two DSBs can form translocation at a very low frequency. A single DSB generated by meganuclease I-SceI or CRISPR-Cas9 could join to any DSB induced by ion irradiation, implying that any two escaped DSBs can form translocation [ 37, 43, 187] . High levels of chromosomal translocations were identified in the presence or absence of C-NHEJ, indicating that both C-NHEJ and MMEJ are involved in translocation formation [43]. Chromosomal translocations may generate new fusion oncogenes or significantly change the expression levels of genes related to cancer, which possibly results in oncogenesis ( Figure 4, bottom left).

During genome editing, DSBs at the target sites are the dominant DSBs and thereby the vast majority of editing outcomes are re-joinings of the two broken ends of the target DSBs. However, other broken ends that occur simultaneously within the edited cells may also have a chance to join with target DSBs to form translocations. These involved DSBs can be categorized into three types: other target DSBs, off-target DSBs, and general DSBs. Correspondingly, the translocations involving these DSBs are referred to as target translocations, off-target translocations, and general translocations, respectively. The target translocation mainly occurs in the multiplex gene editing system and multiple CRISPR-Cas-induced target DSBs join together to induce a high level of chromosomal translocations. The off-target translocations involving DSBs at off-target sites are also dependent on CRISPR-Cas enzymes. As for general translocations, general DSBs induced by various DNA metabolism activities arise randomly in the genome and can also be captured by CRISPR-Cas-induced target DSBs to form chromosomal translocations. These general DSBs may occur in certain physiological processes including V(D)J recombination or class switch recombination in lymphocytes [ 187– 192] , or are triggered by genomic transcription or DNA replication [ 37, 38, 43, 44, 193– 197] . General translocations are distributed widely over the genome with an obvious accumulation at the transcription start site (TSS) [ 38, 43, 44, 187] . Generally, the frequencies of these translocations are in an order of target translocations > off-target translocations >> general translocations.

Several previous reports showed that target chromosomal translocations frequently arose during multiplex genome editing in CAR T manufacturing [ 18, 198– 200] . Chromosomal translocations are also occasionally captured during single-gene editing by many laboratories [ 201, 202] . Using the high-throughput primer-extension-mediated sequencing (PEM-seq), we found that chromosomal translocations occur at a frequency of 1.0%–2.4% in embryonic stem cells (ESCs) and up to 10% in HEK293T during genome editing [ 43, 44] . Cathomen and colleagues also found that chromosomal rearrangements occurred at a ratio of up to 1.6% in edited stem cells [31]. Off-target translocations can be largely suppressed by using high-fidelity Cas9 variants to reduce the break frequency at off-target sites, but the solution to reduce general translocations or translocations among multiple editing loci is still lacking [44]. A recent clinical trial on TCR T therapy indicated that engineered T cells containing translocations among *TRAC-TRBC-PDCD1* remained in the blood at even hundreds of days post-infusion into the patients [18], raising a great concern for these chromosomal abnormalities.

### Chromosomal large deletions

Chromosomal large deletions induced by CRISPR-Cas routinely occur at the target site and range from several hundred bases to megabases, resulting in the loss of a large chromosome fragment around the target site or even the entire chromosome [ 43, 44, 193] . Large deletions arise at a frequency of up to 10% in various human and mouse cell lines based on the sequencing data of PEM-seq or Nanopore DNA sequencing [ 38, 43, 44, 203] ; Bradley and colleagues found that more than 20% of edited mESCs contained deletions more than 250 bp, extending up to 6 kb [41]; Thomas and colleagues found that about 57% edited mouse zygotes contained large deletions up to 2.3 kb [45]. A 3.5 Mb deletion was also identified at the *UROS* loci in HEK293T cells [42]. The mechanism underlying the generation of chromosomal large deletions is not fully understood. We and others found that MMEJ contributes to the formation of large deletions (up to 76.7%) in mESCs and other cells [ 43, 204] . C-NHEJ deficiency could increase the large deletions up to 3 folds [43]. Yet no solutions have been proposed to reduce large deletions during CRISPR-Cas-mediated genome editing ( Figure 4, bottom right).

### Exogenous vector DNA integration

Integration of exogenous DNA originating from vectors or viruses into the genome was another concern of genome editing ( Figure 4, top right). Specifically, the target site is the most frequent integration site [ 44, 180] . György and colleagues found high level of AAV integration (up to 47%) in murine neurons, mouse brain ( *APP ^SW^*, *Mecp2*, and *Dnmt3b*), and moused muscle ( *Dmd*) [180]. As in HEK293T cells, up to 41.3% of edited cells contain vector integration at *RAG1*, *DNMT1*, *EMX1*, *VEGFA*, and *C-MYC* loci, for both Cas9 and its variants [ 43, 44] . Microhomology is widely detected between the integration sites and the integrated fragments, indicating the involvement of MMEJ in vector integration [43]. Additionally, some fragile elements at vectors such as the AAV inverted terminal repeat (ITR) regions can greatly elevate the vector integration level [ 43, 180] .

## Methods for the Detection of Unwanted Editing Byproducts

Many methods have been developed to detect off-target activities of CRISPR-Cas enzymes, both *in vivo* and *in vitro*. The *in vivo* or *ex vivo* methods include LAM-HTGTS, GUIDE-seq, DISCOVER-seq, and PEM-seq, while the *in vitro* methods include but are not limited to Digenome-seq, Dig-seq, CIRCLE-seq, and SITE-seq ( Table 2). These methods have been summarized very well in previous literature [ 28, 35, 38] . Here we focus on the methods to detect other unwanted editing byproducts including chromosomal translocations and large deletions. Quantitative RT-PCR has been widely used to detect chromosomal translocations between two target sites [ 18, 198, 205] , but the resolution is very limited. Whole-genome sequencing or exon sequencing have also been used to identify chromosomal structural variations [ 78, 206] , but these methods are costly and difficult to analyze. Recently, enrichment of target chromatin fragments before sequencing has been introduced to develop several new methods including PEM-seq, LAM-HTGTS, UDiTaS, and CAST-seq (see below for more details). Better enrichment assay or the third-generation sequencing may further facilitate the development of new assays to detect chromosomal translocations or large deletions.

### PEM-seq and LAM-HTGTS

Based on chromosomal translocation capture, both PEM-seq and LAM-HTGTS rely on a Cas enzyme-generated “bait” DSB to capture genome-wide “prey” DSBs *in vivo* [ 37, 38, 207, 208] . The prey-bait junctions are cloned using 1-cycle primer extension for PEM-seq and 80-cycle linear amplification for LAM-HTGTS, followed by ligation with bridge adapters. Subsequent PCR further amplifies the products for next-generation sequencing. Both methods can be used to detect off-target sites that form chromosomal translocations with the bait DSBs as well as large deletions and genome-wide translocations. The LAM-HTGTS was further improved as iHTGTS after optimization of the experimental procedures and the introduction of the random molecular barcode [ 193, 209] . In comparison to LAM-HTGTS and iHTGTS, PEM-seq is a quantitative method which can be used to calculate the frequency of different editing outcomes including vector integrations [ 38, 43] . These methods have been widely applied in mESCs, hESCs, human and mouse primary T cells, various tumor cell lines, and mouse tissues to evaluate the fidelity of Cas9 and Cas12a and their orthologs [ 38, 43, 44, 193, 194] .

### UDiTaS and CAST-seq

UDiTaS, which is based on Tn5 shearing, employs primers on bait and Tn5-introduced adapters to amplify target DSB-involved junctions to identify both chromosomal structural variations and on-target indels [39]. UDiTas was used to identify complex chromosomal rearrangements for *CEP290* and TCR loci in HEK293T cells. A recently developed method CAST-seq employs decoy primers to amplify bait-prey junctions and can be used to detect chromosomal structural variations [31].

## Perspectives

The great improvement of CRISPR-Cas nucleases in clinics shows great potential in the treatment of intractable diseases. Yet DSB is a double-edged sword: off-target damages, chromosomal translocations, large deletions are other non-negligible unwanted editing byproducts that consist of up to 10% of total editing events. The high-fidelity Cas9 variants, especially e *Sp*Cas9(1.1), *Sp*Cas9-HF1, FeCas9, and HypaCas9, are indeed able to effectively reduce off-target activities [44]. However, the solution for other unwanted editing byproducts is still lacking. The decrease of chromosomal translocations or large deletions is usually accompanied by the decline of editing efficiency in previous reports [38]. Given that more than 50% of Cas-induced DSBs are perfect re-joinings and can be cleaved again by CRISPR-Cas until the formation for final indels or degradation of CRISPR-Cas, a Cas enzyme prefers to generate indels rather than perfect re-joinings may narrow the time windows of free DSBs and restrict the generation of various unwanted editing byproducts. On this basis, Cas9TX has been recently developed by our group to greatly reduce chromosomal structural variations by fusing an optimized TREX2 with Cas9. We applied Cas9TX to the next-generation chimeric antigen receptor T (CAR T) engineering and found the levels of deleterious translocations were decreased by tens of folds among multiple targeting sites [210].

Many methods employ inhibitors for DNA repair proteins to change the choice of DNA repair pathways in editing cells [ 141– 143, 158, 159, 211] . However, the perturbation of DNA repair pathways may bring unpredicted editing byproducts that greatly affect genome integrity. For example, the inhibition of C-NHEJ often leads to elevated levels of chromosomal translocations, large deletions, and vector integrations [43]. Moreover, deactivation of p53 in editing cells can also cause genome stability and lead to cancers [ 212– 215] .

## Supporting information

tables
