# Supplementary material for: The origin of unwanted editing byproducts in gene editing: Origin of unwanted editing byproducts in gene editing
Source: Acta Biochim Biophys Sin (Shanghai). 2022 May 25;54(6):767–81. doi: 10.3724/abbs.2022056 (PMC9827802; doi:10.3724/abbs.2022056)
Supplement: tables [file tables.pdf]

Table 1. Application of Cas9 nuclease in clinical therapy*

Target genes	Clinical ID	Therapy	Disease	Stage
TRAC, B2M	NCT03166878, NCT03229876, NCT03166878	CAR-T	B cell leukemia	Phase 1/2
TRAC, TRBC, B2M	NCT04244656, NCT03166878	T cell therapy, CAR-T	Myeloma, B cell lymphoma	-
TRAC, TRBC, PDCD1	NCT03399448, NCT03545815	TCR-T	B cell leukemia and solid tumor	Phase 1
TRAC	NCT03398967	CD19, CD20 or CD22 CAR-T therapy	Relapsed or refractory hematological malignancies	Phase 1/2
	NCT02793856	T cell therapy	Advanced Non-small Cell Lung Cancer	Phase 1
	NCT02863913	T cell therapy	Stage IV bladder cancer	Phase 1
PDCD1	NCT02867332	T cell therapy	Metastatic renal cell carcinoma	Phase 1
	NCT03081715	T cell therapy	Esophageal cancer	Phase 1
	NCT02867345	T cell therapy	Hormone refractory prostate cancer	Phase 1
CD7	NCT03690011	CAR-T	T cell leukemia	Phase 1
CD70	NCT04438083	T cell therapy	Hematologic malignancies and renal cell carcinoma	Phase 1
CCR5	NCT03164135	HSPC therapy	HIV and leukemia	-
BCL11A	NCT03432364, NCT03655678, NCT03745287, CRISPR_SCD001	HSC therapy	Transfusion-dependent β-thalassemia (TDT) and sickle cell disease (SCD)	Phase 1/2
HBB	NCT03728322	HSC therapy	TDT	Phase 1
CEP290	NCT03872479	AAV therapy	Leber congenital amaurosis type 10 (LCA10)	Phase 2
CISH	NCT03538613, NCT04089891	T cell therapy	Metastatic gastrointestinal epithelial cancer	Phase 1/2

*Because of space limitation, only typical methods are cited here.

Table 2. Methods for the detection of byproducts generated by Cas9 *

Method	*In vivo/ in vitro/ In silico*		Assay type	Comment
Cas-OFFinder	*In silico*	Off-target	Sequence alignment	High false positive
CAST-seq	*In vivo*	Chromosomal structural variations, indels	Map translocations with induced DSBs	High-sensitivity; not applicable to limited material
CIRCLE-seq	*In vitro*	Off-target	Sequence cleaved linear DNA from circularized genomic DNA	High-sensitivity; Requires *in vivo* cleavage confirmation
Dig-seq	*In vitro*	Off-target	Whole-genome sequencing for cleaved chromatin	High-sensitivity;
Digenome-seq	*In vitro*	Off-target	Whole-genome sequencing for cleaved naked genomic DNA	High-sensitivity; Requires *in vivo* cleavage confirmation
DISCOVER-seq	*In vivo*	Off-target	Pull down Mre11 binding to broken ends	Narrow time-window (only maps unjoined ends); low resolution
GUIDE-seq	*In vivo*	Off-target	Integrate dsODNs into DSB sites	Unbiased; limited use for blunt-ended DSBs
LAM-HTGTS	*In vivo*	Off-target, chromosomal structural variations	Map translocations with induced DSBs or recurrent DSBs	High-sensitivity; not applicable to limited material
PEM-seq	*In vivo*	Off-target, chromosomal structural variations, indels	Map translocations with induced DSBs or recurrent DSBs	High-sensitivity; not applicable to limited material
SITE-seq	*In vitro*	Off-target	Map broken ends with biotinylated adapters	High-sensitivity; Requires *in vivo* cleavage confirmation
UDiTaS	*In vivo*	Chromosomal structural variations, indels	Map translocations with induced DSBs	Low sensitivity due to no nested PCR

*Because of space limitation, only typical methods are cited here.
